# Small-angle X-ray scattering study of the kinetics of light-dark transition in a LOV protein

**DOI:** 10.1371/journal.pone.0200746

**Published:** 2018-07-16

**Authors:** Katrin Röllen, Joachim Granzin, Renu Batra-Safferling, Andreas Maximilian Stadler

**Affiliations:** 1 Institute of Complex Systems, ICS-6: Structural Biochemistry, Forschungszentrum Jülich, Jülich, Germany; 2 Jülich Centre for Neutron Science JCNS and Institute of Complex Systems ICS, Forschungszentrum Jülich GmbH, Jülich, Germany; Universitetet i Bergen, NORWAY

## Abstract

Light, oxygen, voltage (LOV) photoreceptors consist of conserved photo-responsive domains in bacteria, archaea, plants and fungi, and detect blue-light via a flavin cofactor. We investigated the blue-light induced conformational transition of the dimeric photoreceptor PpSB1-LOV-R66I from *Pseudomonas putida* in solution by using small-angle X-ray scattering (SAXS). SAXS experiments of the fully populated light- and dark-states under steady-state conditions revealed significant structural differences between the two states that are in agreement with the known structures determined by crystallography. We followed the transition from the light- to the dark-state by using SAXS measurements in real-time. A two-state model based on the light- and dark-state conformations could describe the measured time-course SAXS data with a relaxation time τ_REC_ of ~ 34 to 35 min being larger than the recovery time found with UV/vis spectroscopy. Unlike the flavin chromophore-based UV/vis method that is sensitive to the local chromophore environment in flavoproteins, SAXS-based assay depends on protein conformational changes and provides with an alternative to measure the recovery kinetics.

## 1. Introduction

Members of the LOV protein family are blue-light photoreceptors employing LOV domains as photosensory modules, controlling a number of cellular responses like phototropism, chloroplast movement, stomatal opening, regulation of circadian rhythms, photo-induced growth patterns and pigment synthesis [1–5]. LOV domains are structurally well-conserved that typically bind a flavin chromophore (FMN, flavin mononucleotide; FAD, flavin adenine dinucleotide or RF, riboflavin) [6–8]. Upon light absorption, they undergo a metastable covalent bond formation between the C4a atom of the flavin ring and the sulfur of the neighboring cysteine residue [9–12]. The photocycle is thermally reversible and in dark, the FMN-cysteinyl adduct decays to the ground state on a timescale of seconds to days [2, 13–18].

**Fig 1:**
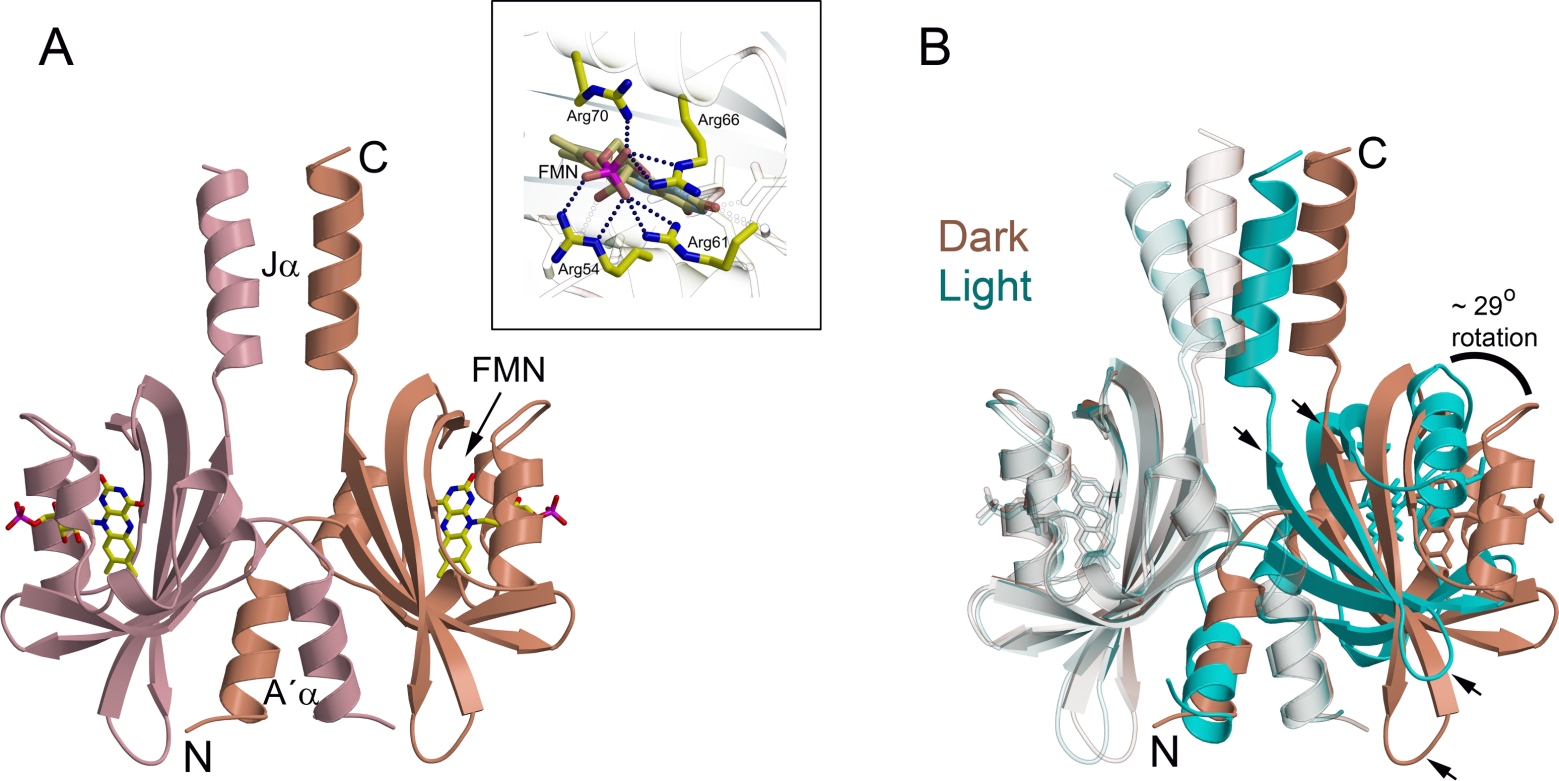
Ribbon representations of PpSB1-LOV dimer. (A) dark state (PDB ID: 5J3W), the N and C termini of a single protein chain are labeled that shows intertwined N-terminus. Protein chains A and B are shown in pink and salmon colors, respectively. Each protein chain is bound to an FMN cofactor shown as stick models (colored by element: carbon, yellow; nitrogen, blue; oxygen, red; phosphorus, pink). The twofold axis runs from top to bottom. Inset to the figure shows the arginine cluster in FMN binding pocket of PpSB1-LOV where dotted lines represent the hydrogen bonds between the FMN and the arginine residues. (B) Superposition of dark (salmon, PDB ID: 5J3W) and light (cyan, PDB ID: 3SW1) states showing structural differences. The LOV core domains of protein chains shown on the left (transparent) were structurally aligned as described previously [28]. In order to superpose the second protein chain (shown on right), a (~ 29°) rotation and translation is required. Arrows indicate the rotation shifts between the respective secondary structure elements.

Structure of LOV proteins are conserved that consist of a core domain with ~110 amino acids that forms a PAS fold composing a central five-stranded antiparallel β-sheet surrounded by helices [19, 20]. In most cases, the core domain is flanked by variable N-terminal or C-terminal helical extensions which are anticipated to play a role in the transfer of signal from the core to the effector domain [2, 21–27]. We recently reported both, dark and fully-adapted light state crystal structures of a short LOV protein PpSB1 possessing core domain flanked by N- and C-terminal helices [14, 28]. A direct comparison of the two structures revealed large structural differences such as ~29° rotation between the two protein chains in the dimer, an altered dimer interface and ~11 Å movement of the C-terminus in Jα helices (Fig 1).

Small-angle X-ray scattering (SAXS) is a well-suited method to study the structure of proteins and macromolecules in solution [29]. Crystal structures of PpSB1-LOV light- and dark-states have already been reported [14, 28], but it is not uncommon that packing effects in crystallography often have a significant effect on domain orientation [30, 31]. Furthermore, the oligomerisation state of a protein in solution can be determined straightforwardly from the measured molecular mass by SAXS. For LOV photoreceptors, the oligomerisation state of light-excited and dark-adapted states is of relevance for the light-activated signal transduction mechanism. For example, in VVD from *Neurospora crassa*, light induction causes dimerization as was previously reported by SAXS [32, 33]. The characteristic biophysical property of LOV photoreceptors is their photocycle, in which the light-state decays to the dark-adapted ground state. SAXS is a unique technique that allows to follow structural transitions between the light- and dark-states of photoreceptor proteins through the dark recovery process [32, 34]. Photoreceptor dark recovery times are typically studied by UV-vis spectroscopy assay. However, one should point out that UV/vis spectroscopy is sensitive to changes in flavin chromophore, whereas SAXS based method is based on changes in protein conformation. Different recovery times measured by UV/vis and SAXS would point out that local changes at the chromophore are not directly translated to structural changes of the protein conformation. To study protein structural transitions as a function of time, time-course SAXS is of advantage over the static structure information obtained from crystal structures that involve lattice-constraints thereby restricting the large-scale movements within protein tertiary structure.

Time-resolved UV/vis spectroscopy was used previously to follow the dark-state recovery of various PpSB1-LOV mutants after photo-illumination [13]. Substitution of the Arg66 to an isoleucine in PpSB1-LOV was reported to accelerate the dark recovery by a factor of 100 to τ_REC_ = 23 ± 1 min in comparison to the wild-type protein (τ_REC_ = 2471 ± 22 min) [13]. Relaxation time-behavior was found to be mono-exponential for wild-type and PpSB1-LOV-R66I indicating that no intermediates are visible during the light- to dark-recovery transition on the probed time-scale by UV/vis [13, 14]. The faster dark recovery of the mutant protein is presumably due to the loss of the salt bridge between Arg66 and the phosphate moiety of the FMN chromophore that was previously observed in the wild-type crystal structure (Fig 1A, inset) [14, 28]. Faster relaxation time of the mutant enabled us to investigate the conformational changes from the light- to the dark-state using SAXS. In the present manuscript, we investigate the blue-light induced conformational transition of a PpSB1-LOV mutant in solution by using time-course and steady-state SAXS experiments.

## 2. Materials and methods

Expression of PpSB1-LOV protein coding genes was performed as described previously [13, 28]. Cells were harvested by centrifugation at 11°C, 4000g for 45 min. Subsequently, ~ 5 g cell pellet was suspended in 150 mL lysis buffer (50 mM NaH2PO4, 300 mM NaCl, pH 8), homogenized using a tissue grinder (Milleville, NJ, USA), and the cells were lysed with a cell disruptor (Constant Systems, Daventry, United Kingdom) three times in a row at 4°C with a maximum pressure of 1.7 kbar. After the separation of cell debris (28,000 g at 11°C for 45 min), the soluble fraction was purified using Ni-NTA Superflow (Qiagen, Hilden, Germany) as per manufacturer’s protocol. The cell free soluble fraction was applied to the column at room temperature, followed by 5 CV (column volume) lysis buffer and 10 CV washing buffer (50 mM NaH2PO4, 300 mM NaCl, 50 mM Imidazole, pH 8). The protein was eluted from the column with 10 CV elution buffer (50 mM NaH2PO4, 300 mM NaCl, 250 mM Imidazole, pH 8). Protein containing fractions were identified using SDS-PAGE, pooled and the buffer was exchanged into storage buffer (10 mM Tris, 10 mM NaCl, pH 7) using PD10 columns (GE healthcare, Freiburg, Germany) following the manufacturer’s protocol. For SAXS measurements all protein samples were applied on size exclusion chromatography (SEC) column Hiload 26/60 Superdex 200 (GE healthcare, Solingen, Germany) prior to SAXS measurements in order to remove any aggregates. The protein solution in storage buffer (10 mM Tris, 10 mM NaCl, pH 7) was concentrated using Vivaspin 20 (Sartorius, Göttingen, Germany). The filtrate was collected and used as control during SAXS measurements.

SAXS data was measured on the beam lines P12 at the PETRA III storage-ring (Hamburg, Germany) [35] and on BM29 at the ESRF (Grenoble, France) [36] using protein from two different protein purifications. The X-ray wavelengths used on P12 and BM29 were 1.24 Å and 1 Å, respectively. Temperature in the sample holder and storage container was 20°C throughout all experiments. Please note that the PpSB1-LOV-R66I protein is stable at this temperature for ≥ 24 hours with no indications of protein degradation or aggregation as analyzed by SEC and DLS (S1 Fig). Used protein concentrations on BM29 were 2.5 and 6.1 mg/mL and 5.2 mg/mL on P12. Total sample volume kept in the storage container of the sample changing robot was up to 1.5 mL for the time-course SAXS experiments on P12. At each time-point of the kinetic SAXS experiment, a fresh fraction of the large sample volume kept in the storage container was used. For the static experiments on BM29 the exposure time was 2 s per collected frame and 10 frames were recorded for each sample. During the kinetic experiments on P12 the exposure time was 100 ms per collected frame and 20 frames were recorded for each individual time point. The radiation dose was thus minimized during the kinetic SAXS experiment that is comparable to the static SAXS experiment. A sample volume of 75 μL was purged continuously through a quartz capillary during a SAXS measurement of each sample at each time point. The individual recorded frames were checked for the absence of radiation damage and the corresponding frames were merged. The scattering contribution of the buffer was subtracted from the merged data sets of the protein solutions. The buffer-subtracted SAXS data were scaled by the measured protein concentrations. Data of the 2.5 mg/mL and 6.1 mg/mL solutions measured under steady-state conditions on BM29 were merged for further data analysis. Time-points were selected to cover the full light-dark relaxation process with an expected light-state fraction of ˜ 1% at the longest measured time-point assuming an exponential decay with a relaxation time of 23 min as determined by UV/vis previously [13].

For the measurements of the protein dark-state, all sample manipulations were performed in the experimental hutch in the dark under red-light conditions. For the steady-state light-state experiments on BM29 the protein solutions were illuminated with a blue-light LED (wavelength 450 nm, radiant power 50 mW, Luxeon Lumileds, Phillips, Aachen, Germany) continuously in the sample storage position and the SAXS experiment was performed under standard light conditions. Concerning the kinetic SAXS experiment on P12 the protein solution in the storage container of the sample changing robot was illuminated with blue-light directly before the experiment for ≥ 60 s. The dark recovery process was followed in the dark with lights being switched off in the experimental hutch. Light of the camera looking at the quartz capillary was turned off for both steady-state and time-course experiments of dark-state measurements.

Data was analyzed and modelled using the programs available within the ATSAS software package [37]: The distance distribution function *P*(*r*) was determined using the program DATGNOM. *Ab initio* models were generated using the program DAMMIN using P2 symmetry. In total 20 *ab initio* models were generated and aligned using the program DAMAVER. The normalized spatial discrepancy (NSD), indicative of the degree of similarity of different bead models, was determined by DAMAVER. For the light-state the mean NSD of all models was 0.544+/- 0.032 standard deviation (SDV) and for the dark-state the mean NSD was 0.556 +/- 0.082 SDV. All models were used for further averaging and filtering by DAMAVER as their NSD were within the range of mean NSD +/- 2*SDV. Those NSD values are indicative of a reliable ab initio model building. The NSD between filtered ab initio models of light- and dark-state is 0.453 indicating comparatively similar overall shapes. The envelope function was determined using the SITUS package[38]. Modelling of unstructured regions and loops was performed using the program CORAL. Here residues that are not resolved in the crystal structures were represented by dummy beads connected to the known crystal structures of the light- and dark-states (PDB ID’s: 3SW1 of the light-state [14] and 5J3W of the dark-state [28]). Ensemble optimization method (EOM) was performed as well using the known crystal structures of light- and dark-state and the known amino acid sequences as input. Standard parameters of EOM were used, which include generation of 10000 different structural conformations that were subjected to the selection of the ensemble distribution.

## 3. Results and discussion

In the first part of our study we investigated the solution structures of PpSB1-LOV-R66I in the fully excited light- and completely relaxed dark-state. The steady-state investigations are prerequisites for time-resolved SAXS experiments that we describe in the second part of the manuscript. Using time-course SAXS we followed the dark-relaxation process of PpSB1-LOV-R66I in real time and compare the obtained relaxation times to the literature value obtained by classical UV/vis assay.

### 3.1 SAXS experiments of PpSB1-LOV-R66I under steady-state conditions

**Fig 2:**
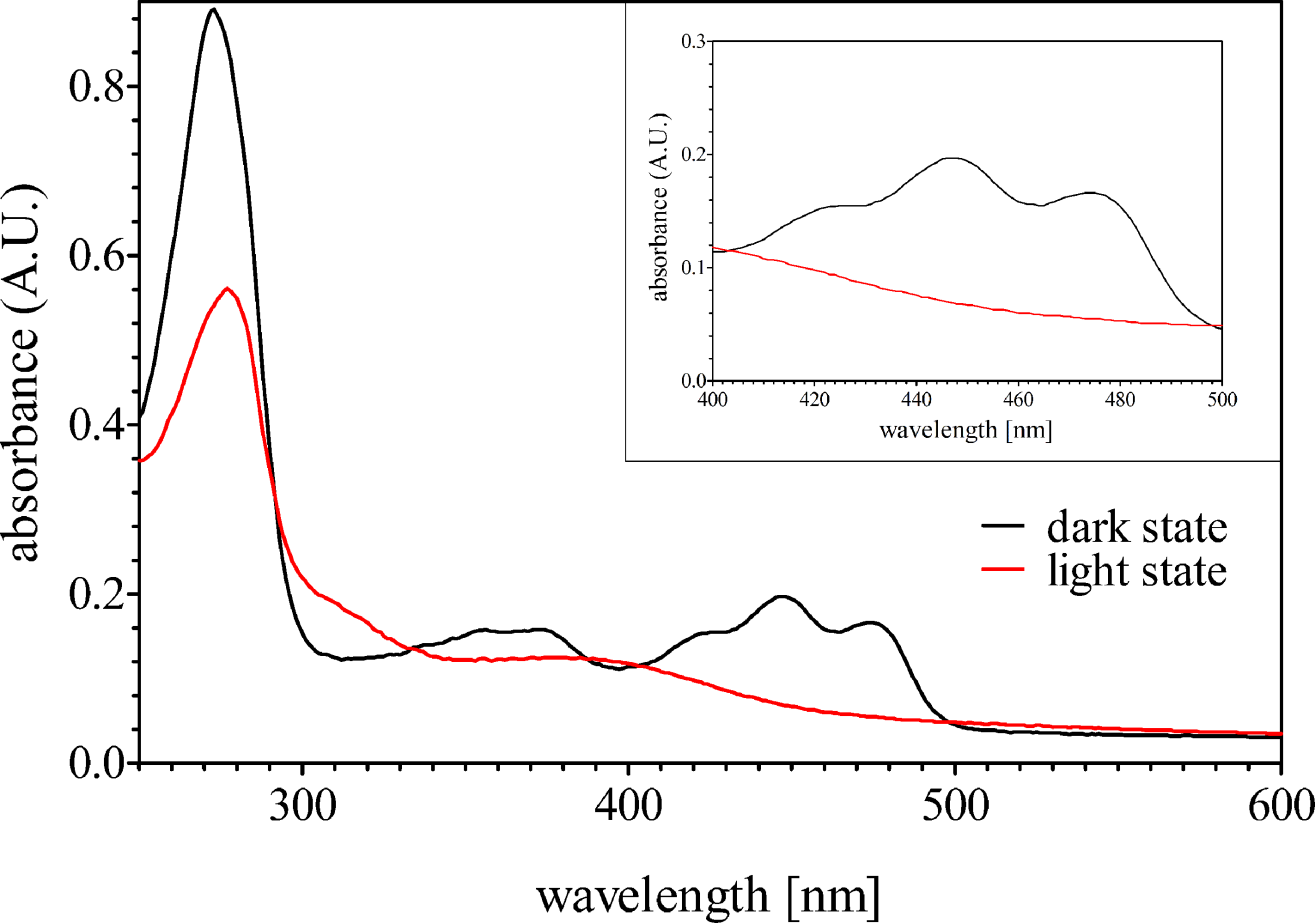
UV/vis spectrum recorded for the PpSB1-LOV-R66I in the light- and dark-states in solution. The inset shows the characteristic absorption maxima at 447 nm after the dark state recovery. The same sample was measured in light- and dark-state. Please note that the difference in absorbance at ˜280 nm is insignificant as this is usually due to the flavin chromophore involved in the adduct formation.

UV/vis spectroscopy of PpSB1-LOV-R66I was measured in the light-excited and dark-adapted states (see Fig 2). The measured spectra show the typical features of PpSB1-LOV-R66I with an absorbance maximum at 390 nm and 447 nm for the light- and dark-states, respectively. The UV/vis experiments thus demonstrate that the protein was in the fully populated states during the SAXS experiment under steady state conditions.

**Fig 3:**
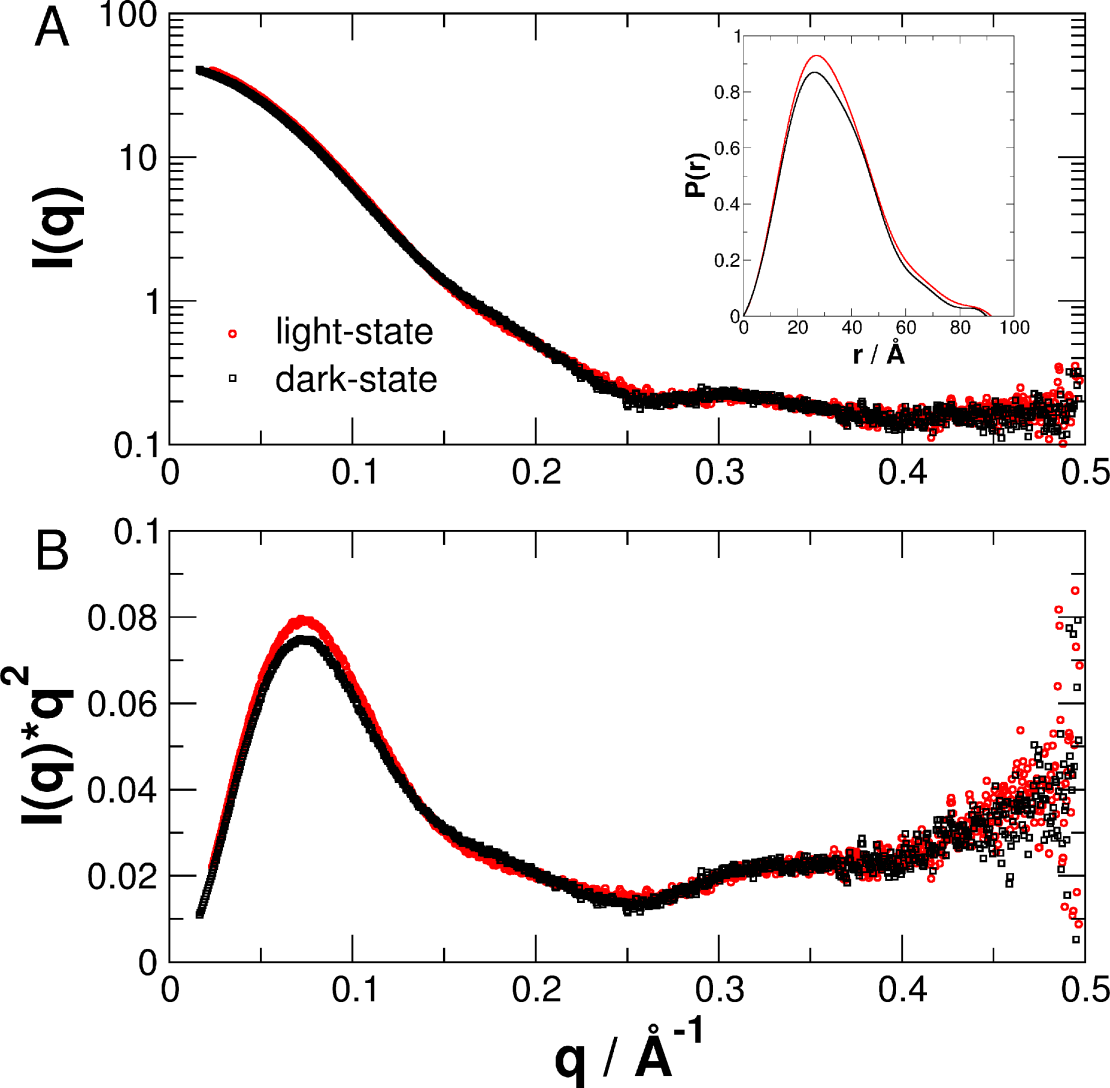
(A) and (B) Small-angle scattering data of PpSB1-LOV-R66I in the light- and dark-states. Distance distribution function *P*(*r*) is shown as inset in (A).

Measured experimental SAXS data *I*(*q*) as a function of the scattering vector *q* of PpSB1-LOV-R66I in the fully populated light-state after blue-light excitation and in the equilibrated dark-state are shown in Fig 3A and 3B.

**Fig 4:**
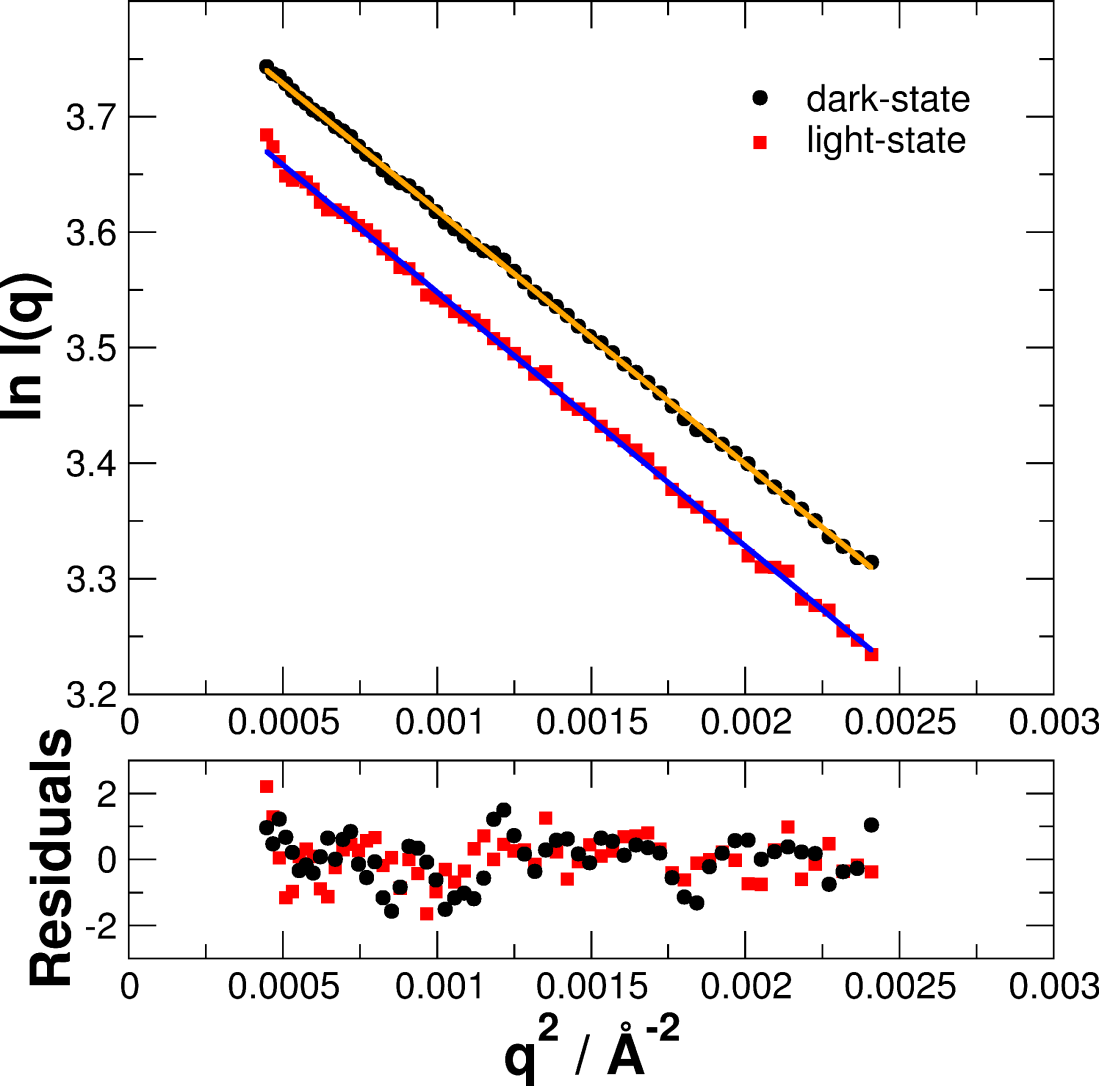
Guinier plots of the merged SAXS data of PpSB1-LOV-R66I in the dark- and light-states. Straight lines are linear fits to the measured data.

The scattering vector *q* is defined in this manuscript as *q* = 4π/λ sin(θ/2) with the scattering angle θ and the X-ray wavelength λ. Distance distribution functions *P*(*r*) were calculated from the measured data, see inset Fig 3A. Direct comparison of the measured SAXS data of the light- and dark-states reveals significant structural differences that are clearly visible in reciprocal space (SAXS data at small *q*-values and the range of 0.15 to 0.25 Å^-1^) and real space (peak shape of the distance distribution function between 20 and 50 Å). The Guinier radius *R*_G_ was calculated using the Guinier approximation in reciprocal space and from the *P*(*r*) distribution in real space. The volume of correlation *V*_c_ was determined according to Rambo and Tainer [39]. Guinier plots of the merged SAXS data are given in Fig 4. The value *Q*_*R*_ *= V*_*c*_^*2*^*/R*_*G*_ was calculated and the molecular mass was determined following Rambo and Tainer as *M*_m_ = Q_R_/0.1231. All physical values of the fully populated light- and dark-states of PpSB1-LOV-R66I based on the merged SAXS data of low and high protein concentrations are summarized in Table 1.

**Table 1:** Physical parameters determined from merged SAXS data. Guinier radii *R*_G_ determined from real and reciprocal space, volume of correlation *V*_c_, ratio *Q*_*R*_ *= V*_*c*_^*2*^*/R*_*G*_, and molecular mass *M*_m_ determined from *Q*_*R*_ of PpSB1-LOV-R66I in the light- and dark-states.

	*R*_G_ (Å) real space	*R*_G_ (Å) reciprocal space	V_c_ (Å^2^)	*Q*_*R*_ *= V*_*c*_^*2*^*/R*_*G*_ *(Å*^*3*^*)*	*M*_*m*_ *(kDa)*
Light-State	25.70	25.73	348.8	4734	38.5
Dark-State	25.61	25.62	343.4	4605	37.4

As pointed out by Rambo and Tainer, both the radius of gyration *R*_G_ and the volume of correlation *V*_c_ are sensitive parameters to detect small conformational changes of proteins in solution. While *R*_G_ values of PpSB1-LOV-R66I are similar in dark- and light-state, the physical parameter *V*_c_ obtained directly from the experimental scattering data indicates that blue-light illumination results in a structural change of the protein as observed previously by X-ray crystallography. The calculated molecular mass of the PpSB1-LOV-R66I monomer from the amino acid sequence is 18.6 kDa (37.2 kDa for the dimer). Therefore, the measured molecular mass *M*_m_ shows that PpSB1-LOV-R66I forms a dimer in solution both in the light- and dark-states.

**Fig 5:**
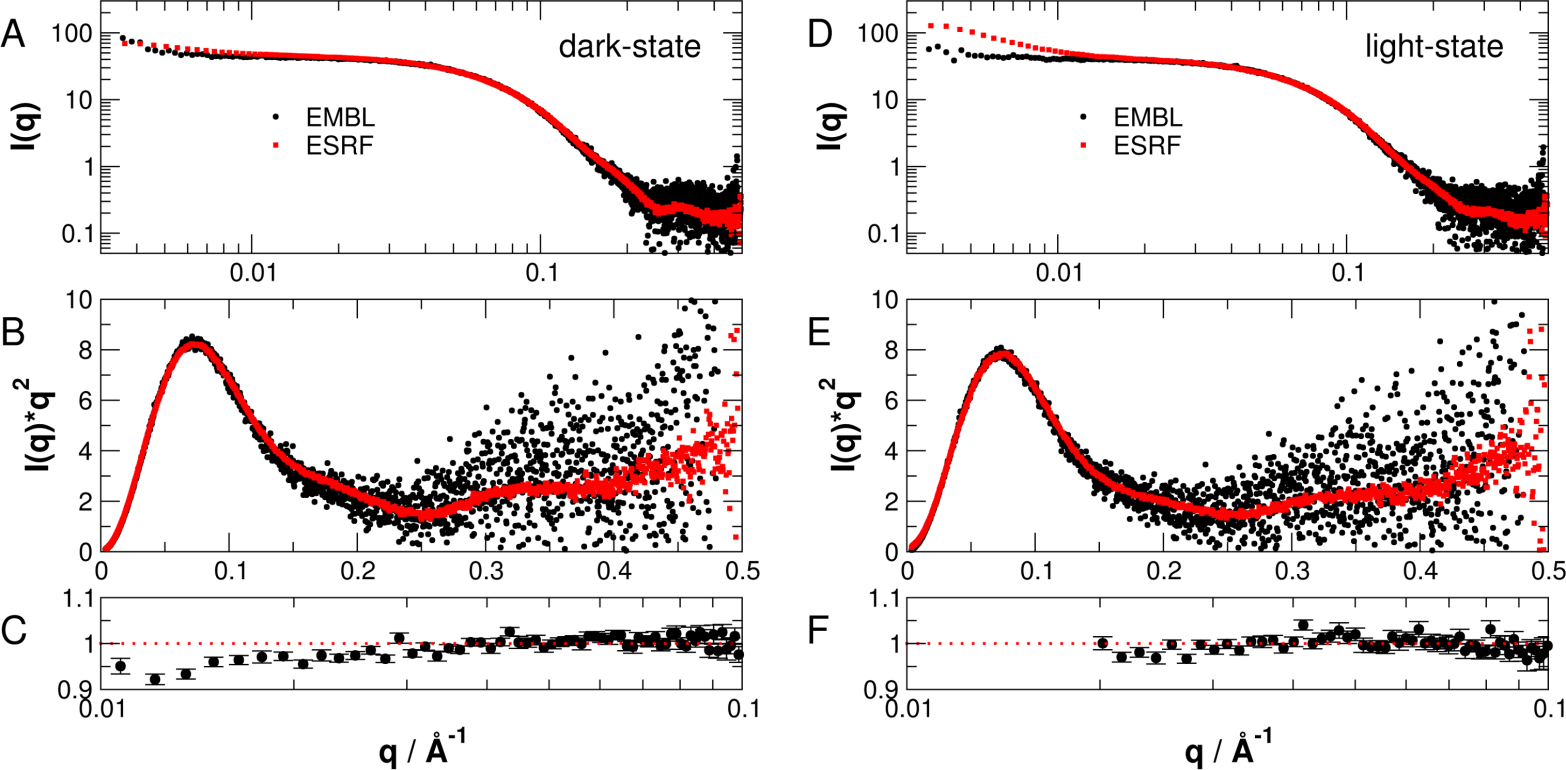
**Comparison of SAXS data of PpSB1-LOV-R66I in fully populated (A, B, C) dark-state and (D, E, F) light-state.** Experimental SAXS data was recorded during two independent experiments on beamlines P12 at EMBL and BM29 at ESRF. (A, D) Log-log plots, (B, E) Kratky-plots of SAXS data. (C, F) SAXS intensities measured on P12 divided by intensities measured on BM29 in the low q-regions that have been used for further analysis.

To validate the reproducibility of our results and to verify the eventual effect of protein aggregation, SAXS data of different protein preparations measured on beamlines BM29 at the ESRF and on P12 at the EMBL are compared in Fig 5. Slight protein aggregation is visible in the SAXS data measured on BM29 at smallest *q*-values, which is not the case for SAXS data recorded on P12 at the EMBL. Protein aggregation is more visible for light- than dark-state sample measured on BM29. To check, whether or not protein aggregation visible in the BM29 data at smallest *q*-values effects the recorded intensities at larger scattering vectors, we directly divided the SAXS intensities recorded on BM29 by the intensities measured on P12 (see Fig 5C and 5F). The division fluctuates around unity within the errors above 0.02 Å^-1^ for light-state and above 0.01 Å^-1^ for dark-state PpSB1-LOV-R66I, which demonstrates clearly that *i*) protein aggregation does not have a significant effect on that *q*-range used for CORAL and EOM modelling as described further below and *ii*) our observations on the structure of light- and dark-state PpSB1-LOV-R66I in solution are fully reproducible.

Crystal structure of the R66I mutant is not available, we thus used the light- and dark-state structures of the wild-type PpSB1-LOV dimer (PDB ID’s: 3SW1 of the light-state [14] and 5J3W of the dark-state [28]) for comparison with the measured SAXS data assuming that the single amino acid exchange at position 66 has no impact on the global structure that is mainly probed by SAXS. Additionally, the dark state crystal structure of the double mutant PpSB1-LOV-R61H/R66I (where an additional arginine 61 was replaced by histidine residue) is essentially same as the wild-type PpSB1-LOV (S2 Fig, S1 Table). The N- and C-terminal residues are structurally disordered and were not included in the final model in the PpSB1-LOV crystal structures (N-terminal 20 amino acids, C-terminal 8–9 amino acids). We modelled these missing residues as dummy atoms representing the unstructured and possibly flexible ends using the program CORAL [37]. It is important to note that CORAL uses a single protein conformation with flexible ends for structural modelling. Thus, CORAL modelling will give good structural reconstructions for a more rigid and less flexible protein that does not possess a large structural heterogeneity. In contrast, CORAL will yield poor reconstructions for systems that are better represented by a broader structural ensemble, as we will see further below.

**Fig 6:**
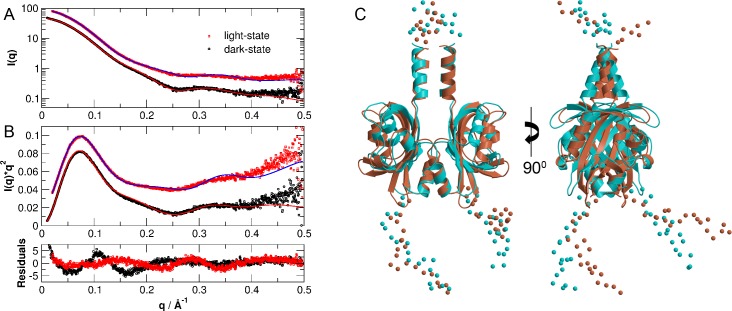
Small-angle scattering data of PpSB1-LOV-R66I in the light- and dark-states and structural models obtained by CORAL. (A) and (B) Experimental data and theoretical scattering curves (solid lines) using the program CORAL of the light- and dark-state crystal structures (PDB ID’s: 3SW1 and 5J3W) including flexible ends that are not seen in the crystal structures. Data are shifted for clarity. The lower panel shows the residuals of the models. (C) CORAL structural models of light (cyan) and dark (salmon) states.

The theoretical scattering curves of the CORAL structure models of the light- and dark-states are shown in Fig 6A and 6B (shown as solid lines) together with the experimental data (shown as filled circles). The theoretical curves are in agreement with the measured SAXS data as demonstrated by the obtained χ-values (a measure of the goodness of fit where lower values indicate a better fit, defined by $=\sqrt{\frac{1}{N}∙\frac{{\left[{I(q)}_{data}-{I(q)}_{model}\right]}^{2}}{{\sigma (q)}^{2}}}$, where *I*(*q*)_data_ represents the measured data, *I*(*q*)_model_ is the model function, σ(*q*) are the errors and *N* the number of data points) of χ = 1.4 for the light- and χ = 2.1 for the dark-state. That observation can also be identified in the residuals (lowest panel of Fig 6), where the residuals of the light-state model fluctuate around zero, whereas the dark-state model of PpSB1-LOV-R66I shows two modulations with minima around 0.5 and 0.15 Å^-1^. This observation shows that CORAL modelling gives a better structural reconstruction for light-state PpSB1-LOV-R66I than for the dark-state. Structural models obtained by CORAL are shown in Fig 6C.

**Fig 7:**
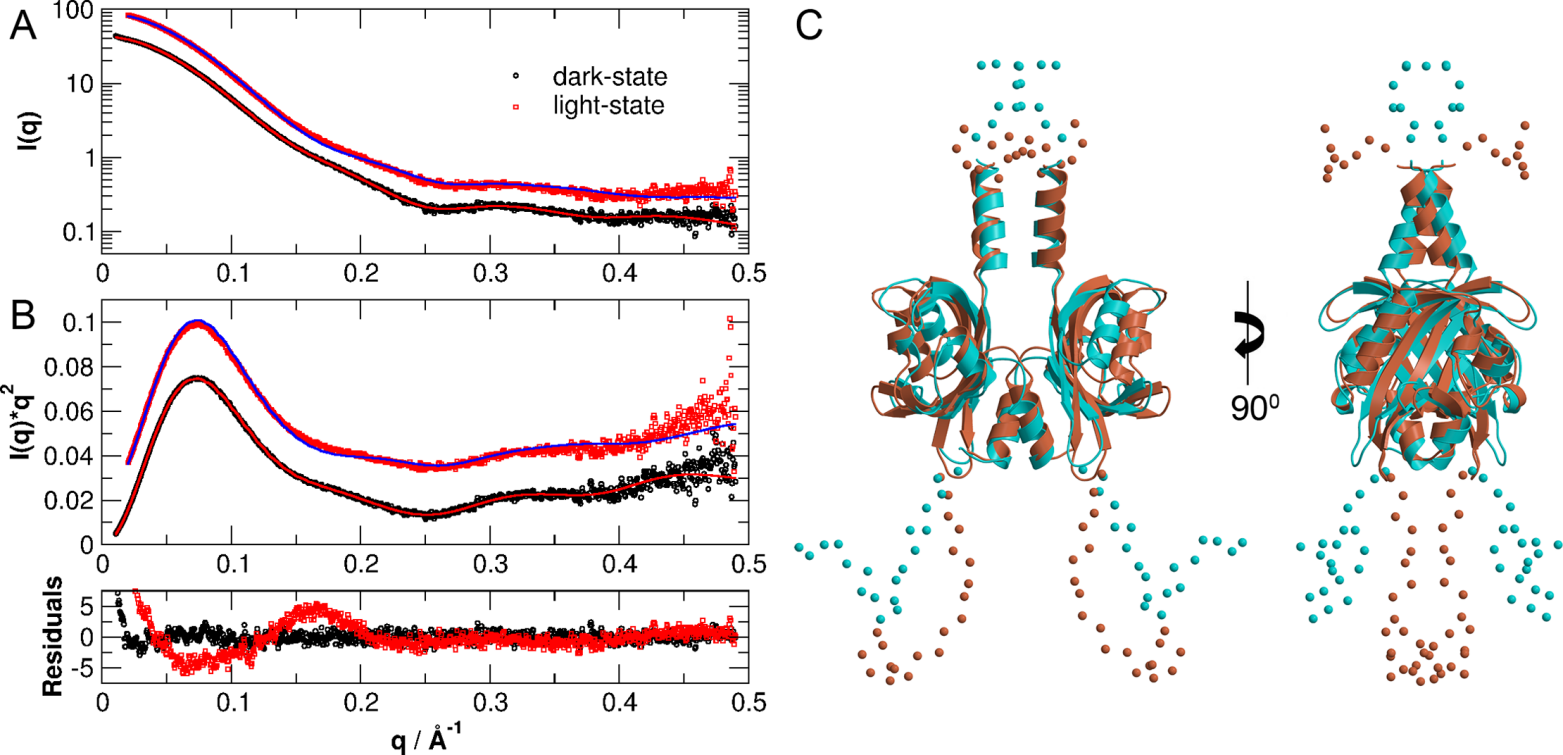
Small-angle scattering data of PpSB1-LOV-R66I in the light- and dark-states structural models obtained by EOM. (A) and (B) Experimental data and theoretical scattering curves (solid lines) using the program EOM of the light- and dark-state crystal structures (PDB ID’s: 3SW1 and 5J3W) including flexible ends that are not seen in the crystal structures. Data are shifted for clarity. The lower panel shows the residuals of the models. (C). EOM structural models of light (cyan) and dark (salmon) states.

To account for flexibility of the unresolved residues, we have performed ensemble modelling using the program EOM as well. The modelling approach of EOM is more applicable for cases, where the protein solution structure is represented by a broader distribution of different conformations. Experimental SAXS data of dark- and light-state PpSB1-LOV-R66I with obtained structural reconstructions using the EOM program are given in Fig 7A and 7B. We obtained χ = 2.37 for the light- and χ = 1.02 for the dark-state. Residuals of EOM modelling are shown in lowest panel of Fig 7. Ensemble modelling of dark-state PpSB1-LOV-R66I by EOM gives an excellent reconstruction of the experimental data: The obtained χ-value is very close to unity and residuals of the structural ensemble representing dark-state SAXS data fluctuate closely around zero and only show a slight upturn at smallest *q*-values that is probably due to a minor influence of protein aggregation. On the other hand, EOM modelling of light-state PpSB1-LOV-R66I yields systematic deviations of the experimental data visible as an oscillation in the residuals and a larger χ-value. Representative structures of light- and dark-state PpSB1-LOV-R66I obtained from EOM modelling are given in Fig 7C.

**Fig 8:**
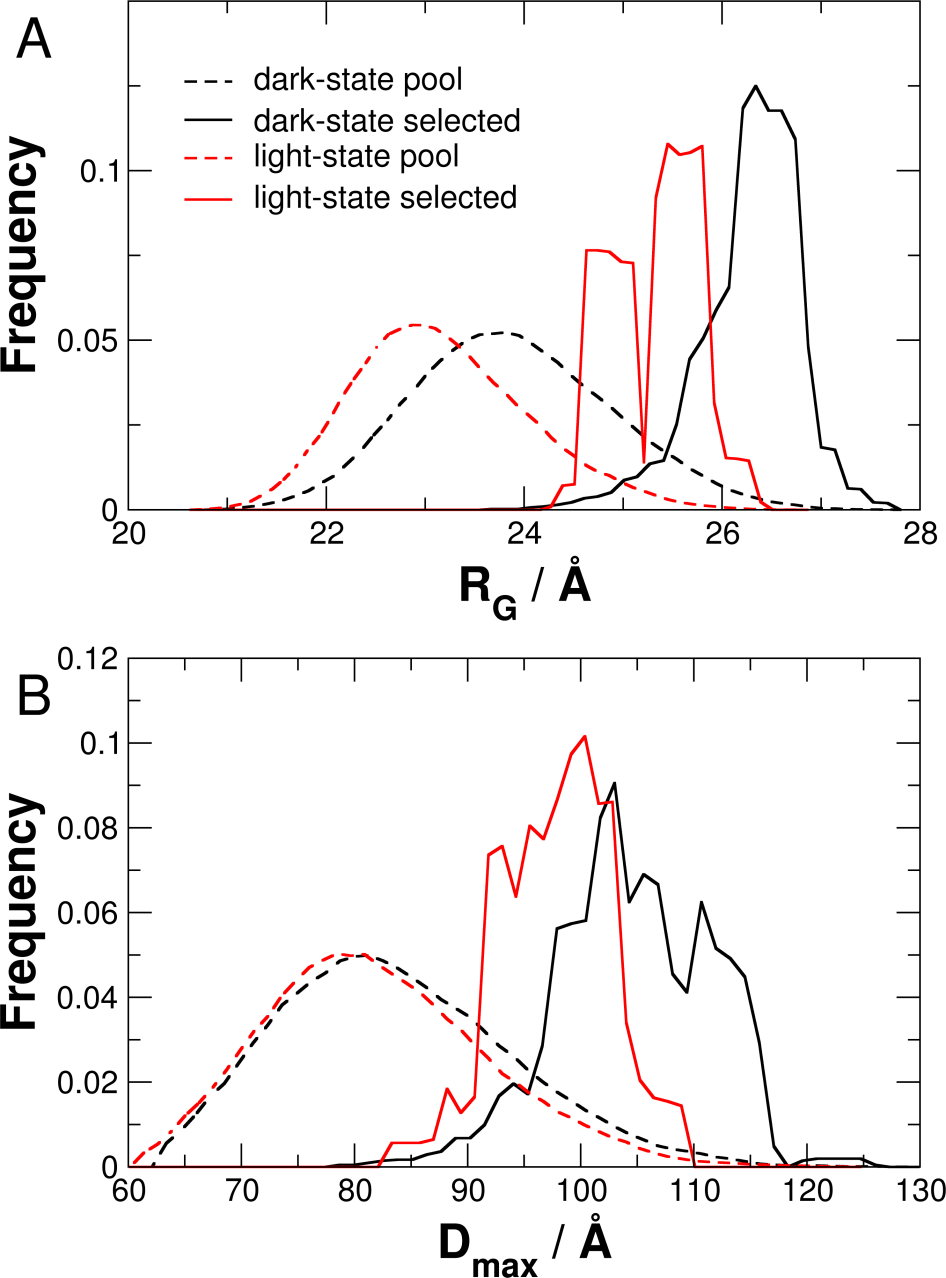
**Distribution of (A) *R***_**G**_ **and (B) *D***_**max**_ **of PpSB1-LOV-R66I in dark- and light-states as obtained by EOM.** Dashed and solid lines show values of generated pool and selected structures, respectively.

The distribution of Guinier radii *R*_G_ and maximal structural dimension *D*_max_ as obtained from the EOM algorithm of dark- and light-state PpSB1-LOV-R66I are given in Fig 8. Visible here as well is that the selected structural ensemble of dark-state PpSB1-LOV-R66I is characterized by a broader distribution than that of light-state PpSB1-LOV-R66I.

In contrast, the corresponding crystal structures without flexible ends reveal a poor fit, resulting in much higher χ-values of 5.9 and 22.3 for light- and dark-state, respectively (see supplementary S3 Fig). As a control, we modelled the missing residues using CORAL and used the dark-state crystal structure to fit the measured SAXS data of the light-state and, correspondingly, the light-state crystal structure to fit the experimental dark-state data (see S4 Fig). We obtained χ = 2.2 for the fit of the dark-state structure against the light-state data, and χ = 4.5 for the fit of the light-state structure against the dark-state SAXS data, which are worse fits than using the correct structural models. If we fit the light-state crystal structure without flexible ends against the dark-state SAXS data and the dark-state crystal structure against the light-state SAXS data, then we obtain χ-values of 10.5 and 4.2, respectively.

**Fig 9:**
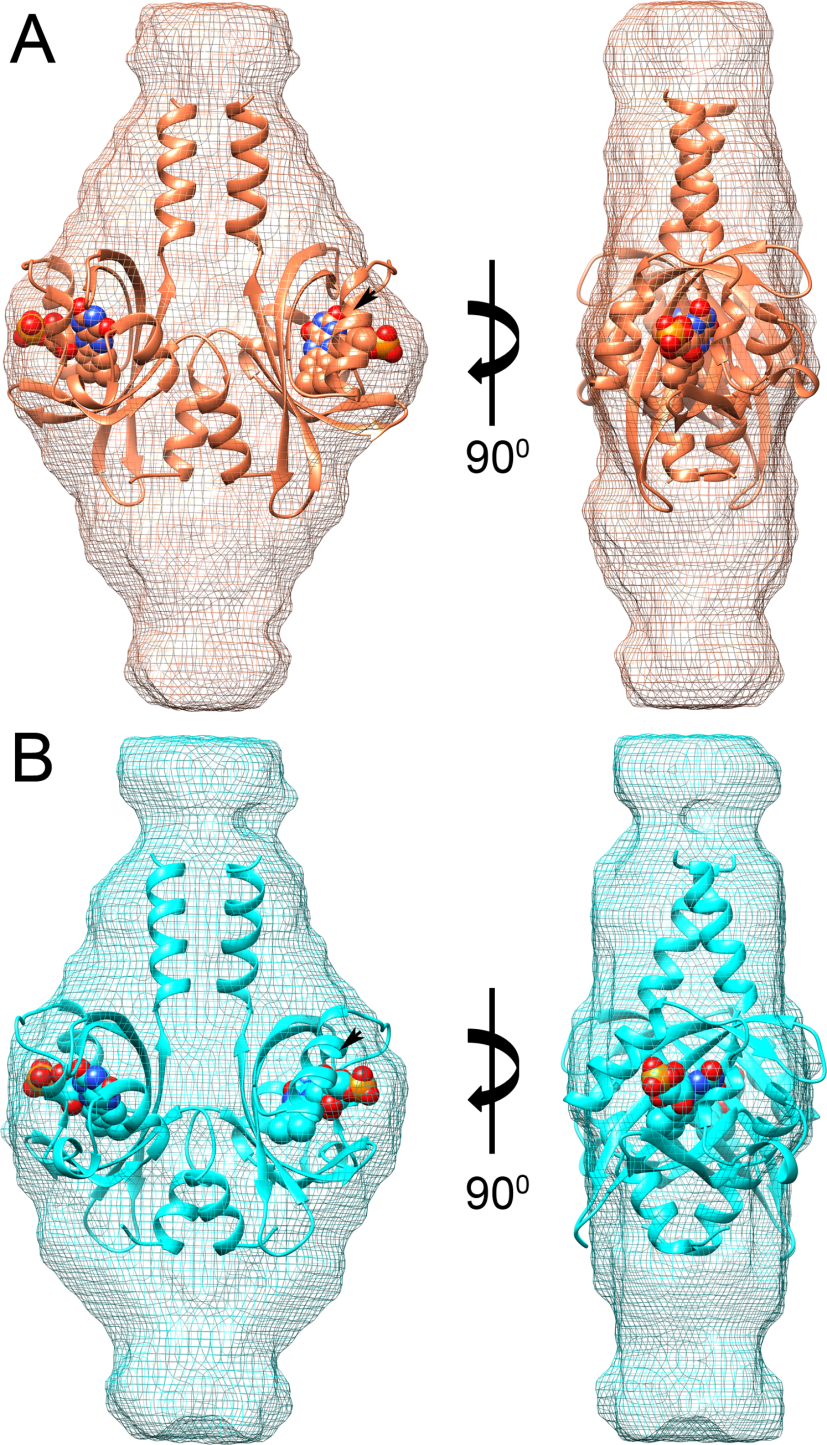
*Ab initio* shape reconstructions of PpSB1-LOV-R66I. (A) Dark (salmon mesh) and (B) light- (cyan mesh) states determined by SAXS. Crystal structures of the dimers of dark- (PDB ID 5J3W) and light (PDB ID 3SW1) are shown as ribbon structures and are aligned in the respective envelopes in cyan and salmon with bound FMN molecules as space filled. Extra density at the top and bottom of the *ab initio* models correspond to residues that are not resolved in the crystal structures. Black arrow heads show the location of Arg66.

We next determined low-resolution envelopes of light- and dark-state structures from the SAXS data by *ab initio* modelling using the program DAMMIN [37] (Fig 9). Due to the limitation of technique, one cannot draw conclusions about structural changes at residue level. However, previously determined crystal structures of both the states of PpSB1-LOV dimers align well within the respective SAXS envelopes as shown in the upper panel of Fig 9.

Note that *ab initio* models primarily serve to visualize measured SAXS data at low resolution. More accurate and reliable information is contained in the physical parameters calculated directly from the experimental data (*R*_G_, *D*_max_ and *M*_m_), and from fits to the measured SAXS data using structural models based on the available crystal structures in reciprocal space as described in detail above (see also Figs 6 and 7).

Our results are thus in agreement with previously reported light and dark state crystal structures of PpSB1-LOV dimers that show large-scale quaternary structural rearrangements. These included ~ 29° rotation of the core domains relative to each other associated with up to ~ 11 Å movement of the C-terminus in Jα helix (Fig 1B) [14, 28]. Extra density observed at the top and bottom of the *ab initio* models accounts for the N- and C-terminal residues that are unresolved in the crystal structures due to high disorder in these regions. Modelling based on two different algorithms (CORAL and EOM) yields further structural information concerning light- and dark-state of PpSB1-LOV-R66I. During CORAL modelling a single structure is refined considering flexibility of the end regions. In contrast, EOM approach considers structural heterogeneity by generation of a large pool of different structures and selection of a structural ensemble that best describes the measured SAXS data. Light-state PpSB1-LOV-R66I is better modelled using the program CORAL than by EOM. On the other hand, dark-state solution structure of PpSB1-LOV-R66I is very well modelled using EOM approach. This indicates different structural flexibility of light- and dark-state: Light-state PpSB1-LOV-R66I is characterized by a single representative structure with restricted structural disorder, while the dark-state is characterized by a larger structural heterogeneity and flexibility of the disordered end regions of helix Jα. This is fully consistent with neutron and NMR spectroscopy studies [28, 40], which reported enhanced picosecond-to-nanosecond dynamics in the C-terminal region of helix Jα in the dark-state as compared to the light-state conformation.

### 3.2 Dark-relaxation process of PpSB1-LOV-R66I investigated by time-course SAXS

In order to gain further information on structural changes occurring during the recovery phase of the photocycle, we performed time-course SAXS measurements of PpSB1-LOV-R66I in dilute solution. The sample was illuminated at the beginning and subsequently experimental SAXS data were recorded as a function of time. Using the knowledge that the light-state of the protein recovers to the dark-state as a function of time the experimental data *I*(*q*,*t*) could be fitted according to

$$I(q,t)=\phi (t)∙{I(q)}_{light}+[1-\phi (t)]∙{I(q)}_{dark}$$ (1)

where *I*(*q*)_light_ and *I*(*q*)_dark_ are the theoretical scattering curves of the light- and dark-state CORAL models obtained from the SAXS experiments under steady-state conditions (see section 3.1 and Fig 6), and ϕ(*t*) is the time-dependent light-state fraction that was used as the only free fit parameter.

**Fig 10:**
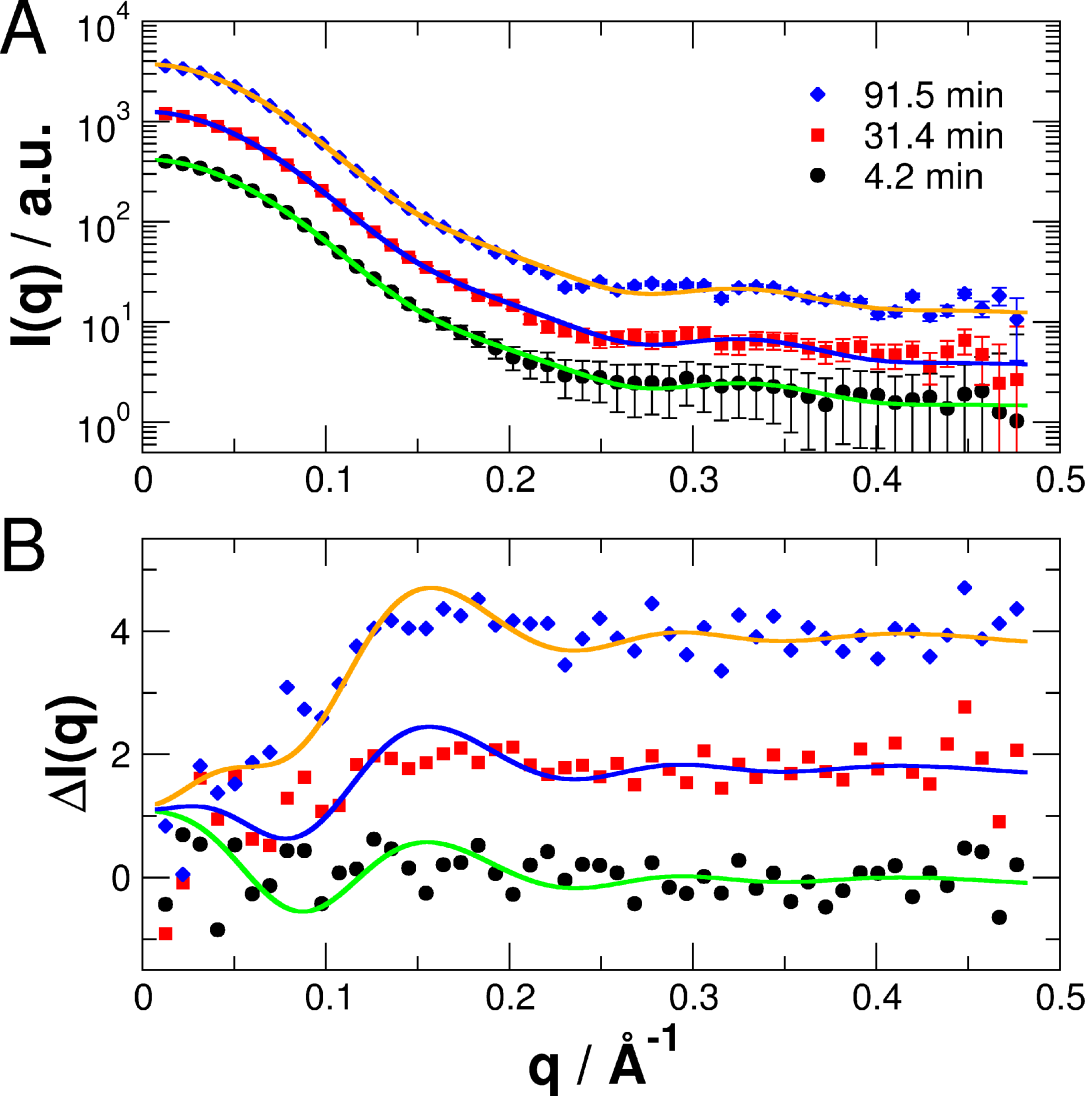
Structural transition from light- to dark-state in PpSB1-LOV-R66I followed by time-course SAXS. (A) Experimental SAXS data measured at selected time-points. Solid lines are fits to the data assuming a two-state population of known PpSB1-LOV-R66I light- and dark-state structures based on the CORAL models with the only free fit parameter ϕ that represents the population of the light-state (Eq 1). Data and fits are shifted for clarity. (B) Difference SAXS patterns Δ*I*(*q*,*t*) of data measured for the time points shown in (A) minus the SAXS pattern recorded at *t* = 0 min. The solid lines are theoretical fits to the data as in (A) based on the CORAL models.

Representative SAXS curves at different time points together with the theoretical fits according to Eq 1 are shown in Fig 10A. The two-component fits yield a good representation of the time-dependent SAXS data with χ-values between 1.02 and 1.10. To illustrate changes of the SAXS pattern as a function of time the difference pattern Δ*I*(*q*,*t*) of SAXS data measured at selected time points minus the SAXS data recorded at *t* = 0 min is shown in Fig 10B together with fits according to the two-state model with the only free parameter ϕ. The theoretical fits shown in Fig 10B are plotted as the difference of the model curves as in Fig 10A minus the theoretical SAXS curve of the CORAL light-state structure.

**Fig 11:**
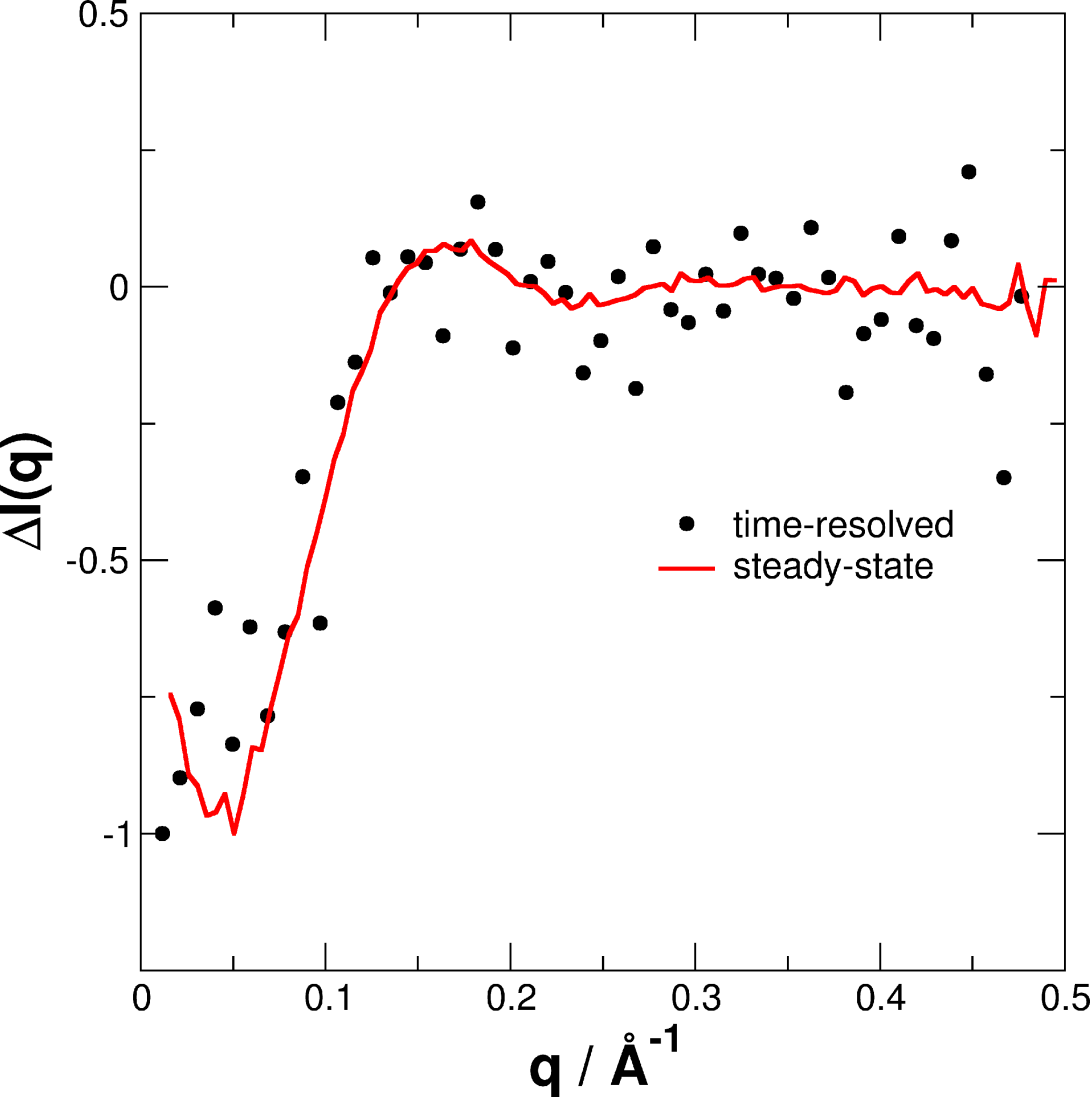
Difference SAXS patterns. Dark- minus light-state measured under steady-state conditions (red line) and the difference of time-resolved SAXS (black circles) data recorded at 98.5 min and 0 min.

The difference SAXS Δ*I*(*q*) pattern of experimental data of the dark-state minus light-state measured under steady-state conditions is shown in Fig 11 in comparison with the longest measured equilibration time at *t* = 98.5 min using Δ*I*(*q*,*t = 98*.*5min*) = *I*(*q*,*t* = 98.5min) - *I*(*q*,*t* = 0min).

**Fig 12:**
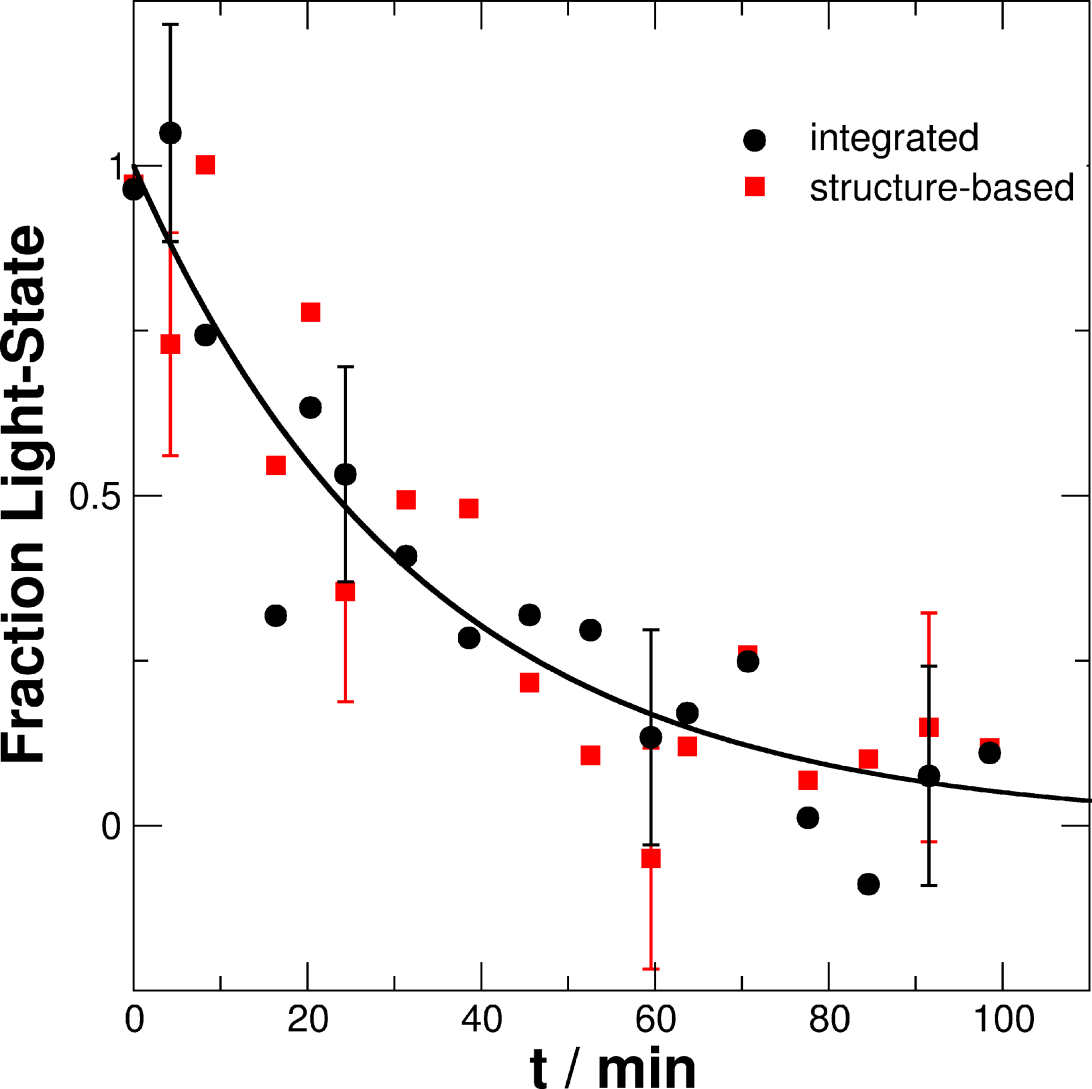
Light-state fraction of PpSB1-LOV-R66I as a function of time. Determined from the integral of Δ*I*(*q*,*t*) up to 0.2 Å^-1^ and a structure-based fit of *I*(*q*,*t*) assuming a mixed population of known light- and dark-states CORAL models. The solid line is a single-exponential fit to the data points of the integral of Δ*I*(*q*,*t*). For clarity error bars are shown for selected points.

The agreement between both, steady state and time-resolved SAXS measurements demonstrate that *i)* SAXS data of the fully populated light- and dark-states show conformational differences, and *ii)* time-resolved SAXS method allows us to follow the recovery process from the fully populated light- to the fully recovered dark-state. The structural transition is clearly visible in the Δ*I*(*q*,*t*) and Δ*I*(*q*) patterns by a loss of intensity in the ranges of 0.02–0.2 Å^-1^ (Figs 10 and 11). Therefore, to follow the recovery process in a model-independent approach Δ*I*(*q*,*t*) was integrated up to 0.2 Å^-1^. Large-scale structural rearrangements are expected to be most visible in this *q*-range. The time behavior of the normalized integral of Δ*I*(*q*,*t*) and the light-state fraction ϕ(*t*) determined from the structure-based fit are shown in Fig 12.

Both the relaxation curves shown in Fig 12 overlap within the errors and could be described well with a single-exponential decay function. We obtained a relaxation time of τ_REC_ = 33.5 +/- 3.6 min and 35.3 +/- 5.2 min for the normalized integral of Δ*I*(*q*,*t*) and the light-state fraction ϕ(*t*), respectively. Within the statistical uncertainty both relaxation times are essentially the same validating our underlying assumptions. We used simple exponential fit, however, caution is warranted as size of the error bars make it inconclusive whether the light-dark transition of PpSB1-LOV-R66I would eventually be better described by a multi-exponential function due to—yet to be found and identified–intermediates.

In the present study, we show structural changes between the fully-adapted light and dark states of PpSB1-LOV-R66I mutant in solution. The calculated curves of the crystal structures of PpSB1-LOV are in excellent agreement with measured SAXS data where flexible residues in the N-terminus and the C-terminus are included in the calculations (Figs 6 and 7). Larger structural flexibility and conformational heterogeneity of the end regions is observed for PpSB1-LOV-R66I dark-state solution structure as compared to light-state solution structure. Furthermore, the structural transition from light- to dark-state in PpSB1-LOV-R66I was followed by time-course SAXS experiments. The measured data could be described by a linear combination of light- and dark-state structures determined from steady-state measurements. The relaxation time (~34 to 35 min) determined is in the same order of magnitude but slower than the relaxation time determined by UV/vis method (23 min) [28]. While SAXS measurements are sensitive to structural changes of the protein molecule at low-resolution, the UV/vis assay is chromophore-based where blue-light induced absorption changes of the FMN molecule in protein environment are monitored. Results of our recovery kinetics indicate that large-scale structural rearrangements involved in the light-induced signaling of PpSB1-LOV-R66I protein and the local changes around the chromophore occur potentially at different rates and appear to be asynchronous. Furthermore, on the covered time-scale of our time-course SAXS experiments no transient intermediates could be observed that have a significantly different quaternary shape than the dark- and light-states. Our experiment, however, does not provide any evidence on structural changes occurring on the time-scale faster than the measured individual time-points. Intermediates might eventually be found using significantly faster time-resolved methods, which is, however, out of the scope of our study. Variation in recovery rates has been observed when implying different techniques. For example, recovery rates for *A*. *sativa* phototropin 1 LOV2 (AsLOV2) domain were previously reported in the range 43.7−70.5 s, 45.5 s and 43.2 s using NMR, CD and visible absorption, respectively [41].

UV/vis spectroscopy assay is a well-established method to determine the photophysical and photochemical properties of LOV proteins which is based on changes in the flavin vicinity, primary steps being the formation of flavin-cysteinyl adduct in the light state, and its spontaneous decay in the dark [9–12]. However, in the context of biological signaling from LOV sensor to the effector domains, it is the change in protein conformation that is of relevance. Recent work of Yee et al. showed that two LOV cysteine less photoreceptors, VVD from *Neurospora* and engineered LOV histidine kinase YF1, were able to induce functional response upon light activation both *in vitro* and *in vivo [42]*. The authors demonstrated that accumulation of the neutral flavin semiquinone radical and hence protonation of the flavin N5 atom is sufficient to trigger signaling response in LOV domain and consequently to the downstream effector domain [42]. These events are linked to conformational changes at tertiary and quaternary level in LOV proteins. Time-course SAXS method presented in this work being independent of the adduct formation can provide information on recovery kinetics of cysteine-less LOV mutant proteins, and likewise on the natural cysteine-less LOV-like regulators which respond to chemical or photoreduction of their flavin cofactors.

## 4. Conclusions

Majority of LOV proteins exist as multidomain proteins with one or more LOV domains fused to the effector domains. So far, structural studies have been focused on the LOV core domain, lacking the effector domains. The knowledge on signal propagation mechanism from the sensor (LOV domain) to the effector domain thus remains insufficient. In a recent report, isolated *Bs*YtvA-LOV domain was characterized by time-resolved X-ray solution scattering [34]. Global structural changes observed upon light-activation of the dimer were identified, such as splaying apart and relative rotation of the two monomers. PpSB1-LOV and its mutant R66I studied here consist of the N- and C-terminal helical elements in addition to the LOV core domain. Consistency of the global changes observed in solution in the current study using SAXS with those observed in the respective crystal structures [28] further supports the signaling mechanism in PpSB1-LOV proteins, where rotation of two protein chains relative to each other causes large movements in the C-terminal Jα helices, relaying the light-activated signal to the associated effector domains. Using site-directed spin labelling (SDSL) and electron-electron double resonance (ELDOR) spectroscopy, Engelhard et. al. recently reported quaternary transitions upon light-activation in the engineered LOV-histidine kinase YF1 where a dominant feature is tilting apart of the two LOV monomers [43]. Based on structural modelling constrained by the ELDOR-derived experimental distance data, the authors proposed that the tilting is accompanied by a slight rotation of the two LOV sensor domains relative to each other. Light-induced structural rearrangements of increased distance and change in relative orientation of the attachment sites for the Jα helices at the C-terminus of LOV core domain are similar to those observed in PpSB1-LOV. The signaling mechanism thus might be preserved amongst dimeric LOV proteins showing similar architecture such as YF1 and LOV protein members of Pseudomonadaceae family [17]. Furthermore, dark recovery kinetics of PpSB1-LOV-R66I using time-course SAXS experiments resulted in relaxation times slower (~34 to 35 min) than the UV/vis method (~23 min). While the relation between light-induced covalent adduct formation and protein conformational changes is well-established in LOV proteins, these results suggest that the recovery of protein conformation and the covalent adduct decay processes might be decoupled where further relaxation processes are possibly involved after the adduct decay.

## Supporting information

S1 Fig Size Exclusion Chromatography (SEC) and Dynamic light scattering (DLS) of PpSB1-R66I. For SAXS measurements, peak fraction of SEC was collected, which was analyzed by DLS. A single peak (middle panel) and a uniform size distribution (lower panel) indicate a homogeneous sample with no aggregation.
(TIF)

S2 Fig Superposition of crystal structures of the double mutant PpSB1-LOV-R61H/R66I (PDB ID: 6GG9 in magenta) and PpSB1-LOV (PDB ID 5J3W in salmon). Each protein chain of the dimer is bound to an FMN cofactor shown as stick models (colored by element: carbon, yellow; nitrogen, blue; oxygen, red; phosphorus, pink). The twofold axis runs from top to bottom. The overall structure of the mutant and the wild type is similar with the a root-mean-square-distance (rmsd) of ~0.68 Å for equivalent Cα atom pairs for all residues in the two dimers.
(TIF)

S3 Fig Measured SAXS data of PpSB1-LOV-R66I in the light- and dark states. Solid lines are theoretical curves of the light- and dark-state crystal structures (pdbs: 3SW1 and 5J3W) lacking the flexible ends.
(TIF)

S4 Fig Measured SAXS data of PpSB1-LOV-R66I in the light- and dark states. Solid lines are theoretical curves of the light- and dark-state crystal structures (pdbs: 3SW1 and 5J3W) including the flexible ends modelled by CORAL. Note that here the theoretical curve of the dark-state crystal structure was fitted against the measured SAXS data of the light-state, and the theoretical curve of the light-state crystal structure was fitted against the measured SAXS data of the dark-state.
(TIF)

S1 Table X-ray crystal data collection and refinement statistics of PpSB1-R61H/R66I-LOV. (PDF)

## Abbreviations

Bs: Bacillus subtilis
CD: circular dichroism
FAD: flavin adenine dinucleotide
FMN: flavin mononucleotide
LOV: Light, oxygen, voltage
NMR: nuclear magnetic resonance
Pp: Pseudomonas putida
RF: riboflavin
SAXS: small-angle X-ray scattering
