# Supplementary figures and images for: Small-angle X-ray scattering study of the kinetics of light-dark transition in a LOV protein
Source: PLoS One. 2018 Jul 16;13(7):e0200746. doi: 10.1371/journal.pone.0200746 (PMC6047819; doi:10.1371/journal.pone.0200746)

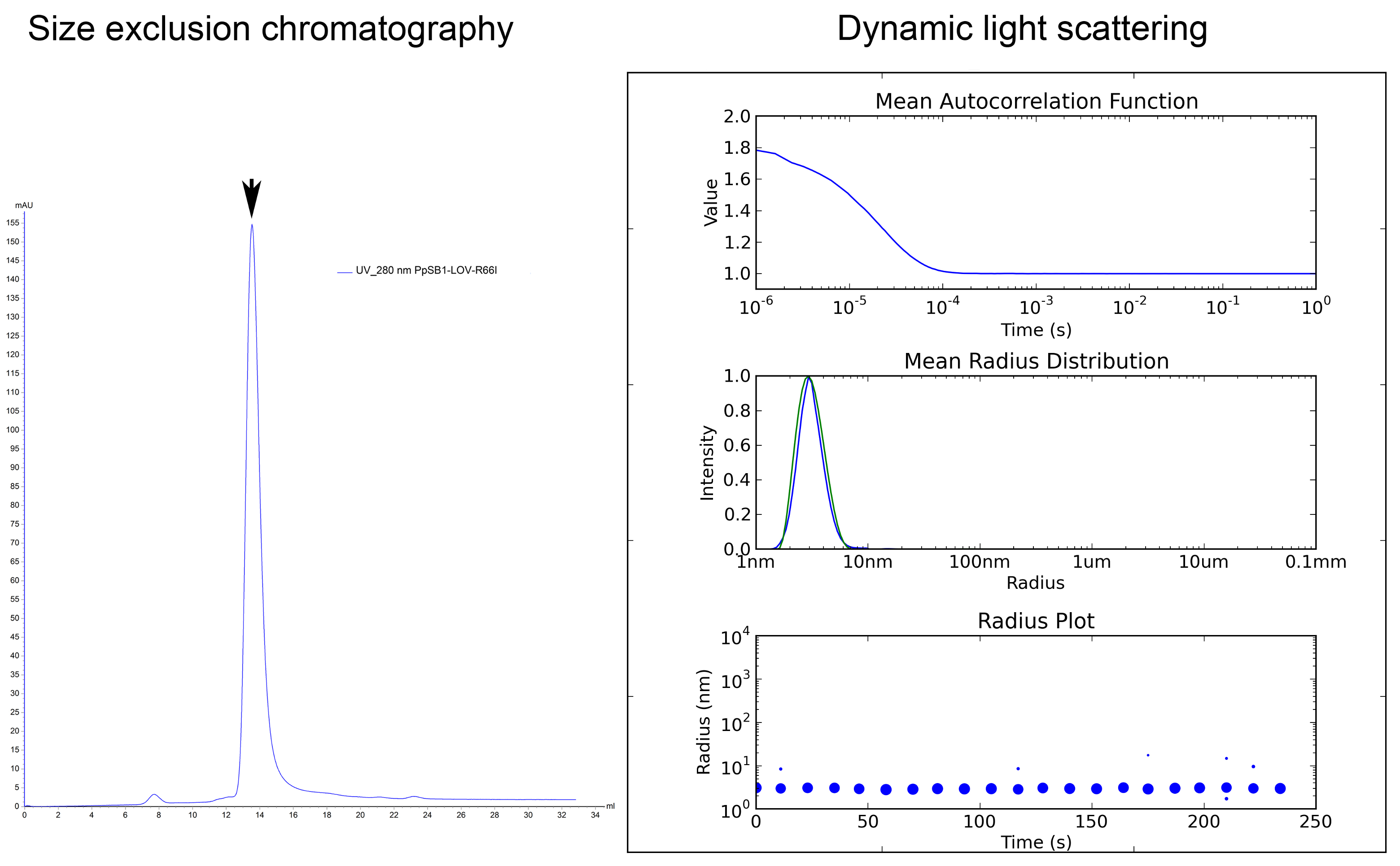

Supplement: S1 Fig — For SAXS measurements, peak fraction of SEC was collected, which was analyzed by DLS. A single peak (middle panel) and a uniform size distribution (lower panel) indicate a homogeneous sample with no aggregation. (TIF) [file pone.0200746.s001.tif]

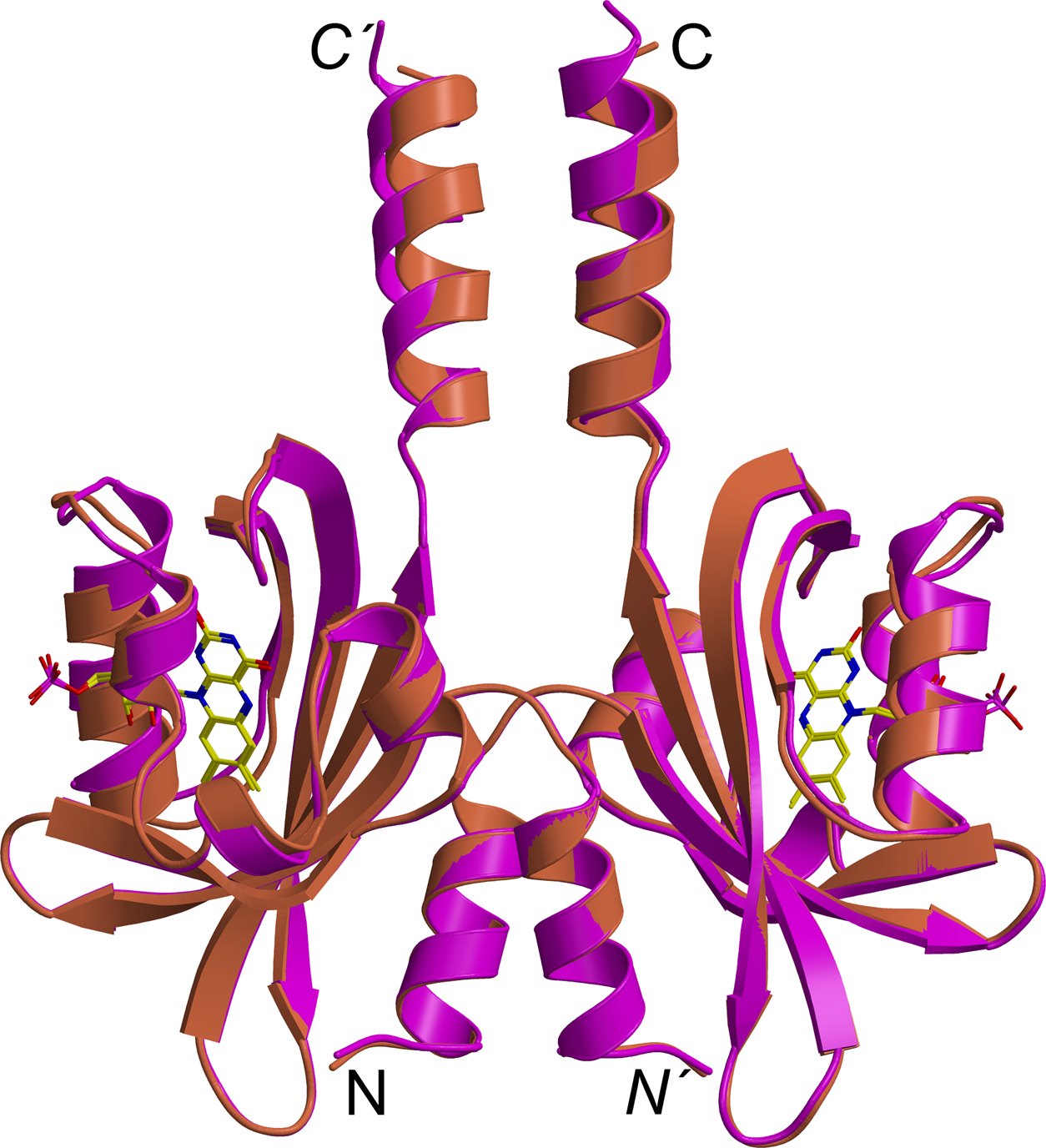

Supplement: S2 Fig — Each protein chain of the dimer is bound to an FMN cofactor shown as stick models (colored by element: carbon, yellow; nitrogen, blue; oxygen, red; phosphorus, pink). The twofold axis runs from top to bottom. The overall structure of the mutant and the wild type is similar with the a root-mean-square-distance (rmsd) of ~0.68 Å for equivalent Cα atom pairs for all residues in the two dimers. (TIF) [file pone.0200746.s002.tif]
