# Supplementary material for: Small-angle X-ray scattering study of the kinetics of light-dark transition in a LOV protein
Source: PLoS One. 2018 Jul 16;13(7):e0200746. doi: 10.1371/journal.pone.0200746 (PMC6047819; doi:10.1371/journal.pone.0200746)
Supplement: S1 Table — (PDF) [file pone.0200746.s005.pdf]

S1 Table: X-ray crystal data collection and refinement statistics of PpSB1-R61H/R66I-LOV.

**X ray data**	PpSB1-LOV-R61H/R66I (**PDB ID: 6GG9**)
Beamline/Detector	ID29, ESRF (Grenoble, France)/DECTRIS PILATUS 6M-F
Wavelength (Å)/Monochromator	λ=0.9772/Silicon (1 1 1) channel-cut
Resolution range (Å)	48.45 - 2.04 (2.10 - 2.04)[x1]
Space group	C 1 2 1
Unit cell a, b, c (Å), α=γ=90°	102.7 76.51 83.28 106.23°
Total reflections	172136 (13667)[x1]
Unique reflections	39334 (3076)x1
Multiplicity	4.4 (4.4)[x1]
Completeness (%)	99.5 (99.7)[x1]
Mean I/sigma(I)	11.2 (1.7)[x1]
Wilson B-factor (Å^2)	37.5
R-merge	0.069 (0.914)[x1]
R-meas	0.078 (1.037)[x1]
**Refinement**	
Resolution range (Å)	44.68 - 2.04 (2.113 - 2.04)[x2]
R-work	0.1962 (0.3045)[x2]
R-free	0.2369 (0.3529)[x2]
coordinate error (max.-likelihood based)	0.29
Number of non-hydrogen atoms	4413
macromolecules	4179
ligands	124
water	110
Protein residues	542
RMS (bonds)	0.008
RMS (angles)	0.93
Ramachandran favored (%)	99
Ramachandran outliers (%)	0.56
Clashscore	4.08
Average B-factor (Å^2)	56.98
macromolecules (Å^2)	57.5
ligands (Å^2)	48.49
solvent (Å^2)	46.81
Number of TLS groups	33

x1, x2 Statistics for the highest-resolution shell are shown in parentheses
